# Anthropometrical and Bioelectrical Impedance Analysis Parameters in Anorexia Nervosa Patients’ Nutritional Status Assessment

**DOI:** 10.3390/medicina55100671

**Published:** 2019-10-03

**Authors:** Joanna Popiołek, Mariusz Teter, Gustaw Kozak, Tomasz Powrózek, Radosław Mlak, Hanna Karakuła-Juchnowicz, Teresa Małecka-Massalska

**Affiliations:** 1 Department of Human Physiology, Medical University of Lublin, 20-080 Lublin, Poland; mariusz.teter@umlub.pl (M.T.); tomasz.powrozek@umlub.pl (T.P.); radoslaw.mlak@umlub.pl (R.M.); 2 I Department of Psychiatry, Psychotherapy and Early Intervention, Medical University of Lublin, 20-439 Lublin, Poland; gustaw.kozak@umlub.pl (G.K.); hanna.karakula-juchnowicz@umlub.pl (H.K.-J.)

**Keywords:** phase angle, starvation, undernutrition, body mass index, bioelectrical impedance analysis

## Abstract

*Background and Objectives:* Body mass index (BMI) is still the only recommended measurable nutritional status assessment parameter in anorexia nervosa (AN). The aim of this study was to measure other anthropometrical and bioelectrical impedance analysis (BIA) parameters in AN patients and to evaluate their nutritional status assessment value. *Materials and Methods:* The 46 AN female patients were examined at the beginning of hospitalization and followed-up in three measurements (in 6 ± 2 weeks’ intervals). Anthropometrical assessment was based on BMI, circumferences of arm, calf, thigh, hips, waist, their ratio (waist-to-hip ratio (WHR)), and a skinfold test over biceps and triceps muscle, under the scapula, over the hip, and 2 cm from the umbilicus. The BIA parameters included phase angle (PA), membrane capacitance (Cm), and impedance at 200 kHz, and a 5 kHz ratio (Z_200/5_). *Results:* In the 1st measurement, BMI correlated with all anthropometric and BIA parameters (*p* < 0.05). For BIA parameters, the correlation included arm circumference and WHR (*p* < 0.05). In the follow-up, significant changes were observed in BMI and all BIA parameters. The correlation between BMI and all BIA parameters was present in the 2nd and 3rd measurements (*p* < 0.05). In the 4th measurement, BMI correlated only with Cm (*p* = 0.0114). Comparison of BIA parameters according to the state of starvation (BMI < 16.0 kg/m^2^) revealed that all studied BIA parameters were characterized by statistically significant sensitivity and specificity in the detection of this condition (*p* < 0.05), except PA in the 4th measurement (*p* = 0.2099). *Conclusions:* Selected BIA and anthropometrical parameters could be used for AN patients’ assessment. The study confirmed dynamic changes of BIA parameters during the follow-up. They could be useful in the detection of the state of starvation.

## 1. Introduction

Anorexia nervosa (AN) is a complex eating disorder combining mental and nutritional status abnormalities. In the course of the disease, physicians deal with both of these aspects. Apart from a professional psychiatric assessment, current guidelines (International Statistical Classification of Diseases and Related Health Problems (ICD-10)) focus on body mass index (BMI) as a diagnosis criterion (17.5 kg/m^2^ or lower) [1]. Additionally, it is used as a measurable parameter for treatment progress assessment. Furthermore, according to the Diagnostic and Statistical Manual of Mental Disorders (DSM 5) classification, there are four AN types depending on BMI value: mild (BMI > 17 kg/m^2^), moderate (BMI 16.0–16.99 kg/m^2^), severe (BMI 15.0–15.99 kg/m^2^), and extreme (BMI < 15.0 kg/m^2^) [2]. Similarly, the WHO extended the BMI classification to distinguish the underweight (BMI 16.0–18.5 kg/m^2^), severely underweight (BMI 15.0–15.99 kg/m^2^), and very severely underweight (BMI < 15.0 kg/m^2^) among the malnourished [3]. However, more measurable parameters are reported that can be used for undernutrition assessment in this group of patients. They include anthropometrical and electrical parameter measurements [4,5,6].

Anthropometrical assessment is based on measurements of circumferences (arm, calf, thigh, hips, and waist) or their ratio (waist-to-hip ratio (WHR)) and a skinfold test in specific regions (over biceps and triceps muscle, under the scapula, over the hip, and 2 cm from the umbilicus). Anthropometrical measurements also include BMI calculation. Those are noninvasive and do not require the use of electronic devices. Furthermore, on the basis of these values, fat mass can be secondarily assessed using the formulas.

Electrical parameters can be measured with bioelectrical impedance analysis (BIA). This noninvasive method enables the assessment of the electrical properties of tissues. It is based on running a series of alternating electric currents through the body. These signals interact differently with body cells and fluids, transmitting the potential difference (voltage) back to the BIA device. The measurement can be taken at different electric current frequencies. The BIA parameters in this spectrum vary depending on the frequency used. The resulting data is given in Ohms as impedance (Z), which is a combination of resistance (R) and reactance (Xc). The Z_200/5_ parameter, which is impedance at 200 kHz, and a 5 kHz ratio can be used for nutritional status assessment and monitoring.

Phase angle (PA) is the relationship between the electrical current passing through the body and the potential difference invoked by this current across body tissue. PA is commonly accepted as a prognostic indicator of morbidity and mortality. This has been demonstrated in various population groups including oncology, HIV, liver cirrhosis, heart failure, hemodialysis, and sepsis patients [7,8,9,10,11,12,13,14,15,16,17,18]. Furthermore, there are papers that prove its significance in AN patients’ nutritional status, treatment sufficiency, and recovery assessment [19,20,21,22,23,24,25,26].

Due to their structure and properties, cells act as capacitors and generate membrane potential. Healthy cell membranes are poor conductors but good capacitors. That is why another parameter, cell membrane capacitance (Cm), reflects cellular membrane integrity [27,28]. Malnutrition reduces cellular membrane mass and integrity and promotes shifts in fluid balance. Destruction of cell membranes secondary to poor nutritional status impairs their capacitance properties. That is why low Cm could be another BIA malnourishment marker.

Furthermore, by means of the analysis of tissue properties, BIA can identify them and provide reliable information about body composition, e.g., about fat mass.

**Figure 1:**
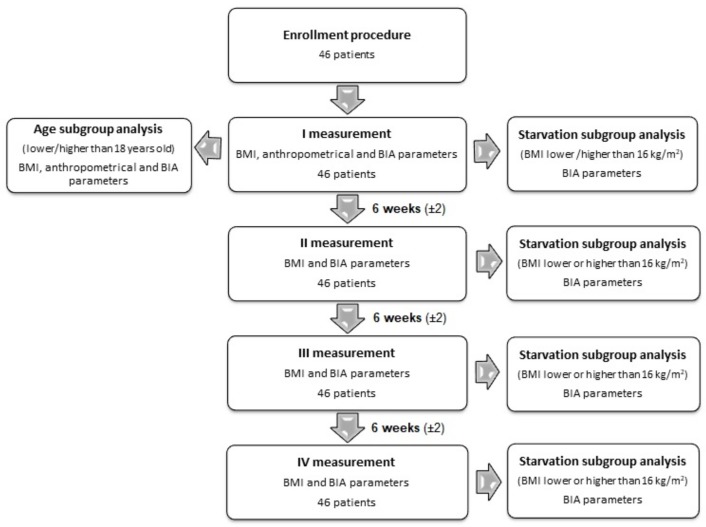
Study design. BMI; body mass index, BIA; bioelectrical impedance analysis.

The primary purpose of this study was to assess the anthropometrical and electrical parameters and their correlation in AN patients at the beginning of the treatment and during follow-up. The secondary purpose of the study was to evaluate the usefulness of applying the BIA parameters in undernutrition level assessment. For this purpose, the results were grouped by BMI value lower or higher than 16 kg/m^2^, representing the state of starvation. Then the sensitivity and specificity of BIA parameters for the detection of the state of starvation were statistically assessed. The BMI value was set at 16 kg/m^2^ because it is the borderline between moderate and severe AN [2]. This value is identical for the borderline between the underweight and severely underweight [3]. The study design is presented as a flow diagram in Figure 1.

## 2. Materials and Methods

The study included 46 female patients (mean age: 17.38 ± 4.99 years) diagnosed and hospitalized due to AN at the I Department of Psychiatry, Psychotherapy and Early Intervention of the Medical University of Lublin in the time period between May 2015 and April 2018. It was based on a detailed examination at admission followed by three measurements in 6 (±2) week intervals as the follow-up. All patients hospitalized at that time met the inclusion criteria and were enrolled in the study. All the study participants have completed full follow-up. The study protocol was approved by the local Bioethical Commission of the Medical University of Lublin (consent no. KE-0254/232/2014). Before being enrolled in the study, all patients signed the informed consent form.

Inclusion criteria:

1) Written informed consent (signed by participant or/and a parents or legal guardian);
2) Female gender;
3) In- and outpatients;
4) Fulfilling diagnostic criteria for AN according to the ICD-10 [1];
5) Secondary amenorrhea.

Exclusion criteria:

1) Life-threatening conditions during AN;
2) Presence of another psychiatric disorder before AN diagnosis (including organic brain dysfunction, cognitive disability, addiction), except a specific personality disorder;
3) Diagnosis of gastrointestinal disease which could affect diet or dietary habits (inflammatory bowel disease, Crohn’s disease, colitis ulcerosa, irritable bowel syndrome);
4) Epilepsy;
5) Peripheral venous insufficiency occurrence (e.g., congestive heart failure, cirrhosis, and nephritic syndrome);
6) Endocrine disorders (e.g., diabetes mellitus, diabetes insipidus, and hypo- or hyperthyroidism) before AN diagnosis;
7) Body core temperature above 38 °C;
8) Creatine use at the time of inclusion in the study or 4 weeks before enrollment;
9) Having medical implants (e.g., cardiac devices or metal joint replacements).

Anthropometrical measurements included BMI; WHR; arm, calf, thigh, hip and waist circumferences; and a skinfold test over biceps and triceps muscle, under the scapula, over the hip, and 2 cm from the umbilicus. BMI was calculated using the (weight (kg))/(height (m))^2^ formula. Circumferences were measured by a qualified physician at listed locations three times using body tape measure. The results were given in centimeters with 0.5 cm accuracy. Then the mean values used for later statistical analysis were calculated. The skinfold test was conducted analogically in listed locations with a Baseline ^®^ Skinfold Caliper (White Plains, New York, USA) three times. The results were given in millimeters with 1 mm accuracy. WHR was calculated using the waist circumference (cm)/hips circumference (cm) formula.

BIA measurements were conducted with ImpediMed bioimpedance analysis SFB7 BioImp v1.55 (Pinkenba, Brisbane, Qld 4008, Australia) in triplicates. Then the mean values were used for statistical analysis. The provided system used four self-adhesive surface electrodes connected to the analyzer. Electrodes were placed on the right wrist and right ankle carefully after washing the skin. The measurements were taken in the horizontal position with limbs resting loosely at 30–45 degrees to the body. Before the examination, patients had to lie in position for 5 min and were not allowed to drink, eat, or make any physical effort in the preceding three hours, in accordance to producers’ procedure recommendations. The resulting data were based on Z, which is a combination of R and Xc. Resistivity of cells demonstrates resistance, whereas reactance is the result of the electric capacity of the cell membranes. Resistance causes the voltage drop, while reactance causes the phase shift of the applied alternating current. The phase shift is given as the time difference between voltage and current signal waves. The measurement was conducted at different frequencies: 50 kHz for PA and Cm as the recognized standard, and 5 kHz and 200 kHz for Z. The Z_200/5_ parameter was defined as Z at 200 kHz, and a 5 kHz ratio was then calculated.

The collected data were analyzed using the Statistica 13 software (StatSoft ^®^, Kraków, Poland). To achieve the primary purpose of the study we performed the following statistical analysis as described below. Changes in the weight, BMI value, and selected BIA parameters (Cm, PA 50 kHz, and Z_200/5_) over time were evaluated using the ANOVA Friedman test (test for 3 or more dependent variables, measurement of changes in subsequent months of observation). Correlation between weight, BMI, Cm, PA 50 kHz, Z200/5, and selected anthropometric parameters (skinfold biceps muscle, skinfold triceps muscle, skinfold under the scapula, skinfold over the hip, skinfold 2 cm from the umbilicus, arm circumference, thigh circumference, calf circumference, waist circumference, hip circumference, and WHR) were assessed using the Spearman rank correlation test (nonparametric test for variables with non-normal distribution). Moreover, the same test was used for the evaluation of correlation between weight as well as BMI and BIA parameters (Cm, PA 50 kHz, Z200/5) in subsequent months of observation. Comparison of the level of selected variables (BIA parameters: Cm, PA 50 kHz, Z200/5) in subsequent months of observation (according to a BMI status below or above 16.0 kg/m^2^) was carried out using the Mann–Whitney U test (nonparametric test used to compare 2 variables with non-normal distribution). The same test was used for the comparison of the level of selected variables (body composition as well as the above-mentioned anthropometric and BIA parameters) according to the age of patients (below or above 18 years old). To achieve the secondary purpose of the study, we performed the following statistical analysis as described below. The usefulness of selected BIA parameters in the detection of patients in the state of starvation (for the purpose of the analysis patients were divided according to BMI, below or above 16.0 kg/m^2^) was analyzed using the ROC curve analysis (for each of the possible cut-off points, sensitivity (true positive results divided into the sum of true positive and false negative results) and specificity (true negative results divided into the sum of true negative and false positive results) were calculated), and then the results obtained were marked on the graph with an ROC curve. Based on the ROC curve analysis, the optimal cut-off point with the best sensitivity and specificity was selected for each parameter tested. Results with a *p*-value of <0.05 were considered as statistically significant.

## 3. Results

The study group consisted of 46 women between 11 and 25 years of age. 34.79% patients were over 18 years old whereas the others were adolescents. The mean BMI and WHR values noted in the study group were 16.05 and 0.73, respectively. Detailed characteristics of the patients are presented in Table 1. Subgroup analysis revealed that younger patients (<18 years old) had significantly lower values of fold triceps muscle (8.50 vs 5.00 mm; *p* = 0.0225) as well as higher values of Cm (0.89 vs 0.65; *p* = 0.0198). A comparison of selected anthropometrical and BIA parameters according to the age of patients is presented in Table 2.

### 3.1. Changes in BMI and Selected BIA Parameters in Subsequent Months of Observation

**Figure 2:**
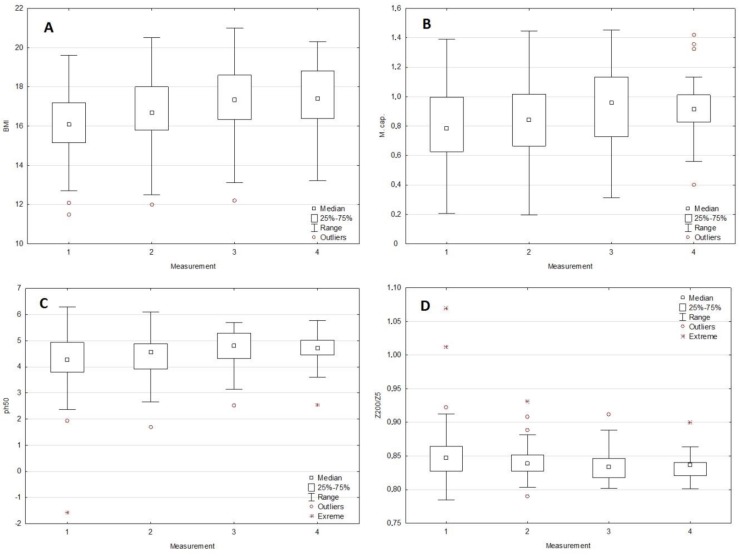
Changes in BMI (**A**) and selected BIA parameters:membrane capacitance (**B**), phase angle 50 kHz (**C**) and Z_200/5_ (**D**) in the subsequent months of observation (measurements I to IV).

There were significant changes between the I and III or IV measurements, both in the case of BMI (*p* = 0.0366) as well as selected BIA parameters (Cm (*p* = 0.0109), PA (0.0075), and Z_200/5_ (*p* = 0.0297)). Both for Cm and PA, similarly as in the case of BMI, an increase in values was observed in the subsequent months of observation. In the case of the Z200/5 parameter, an inverse relation (decrease in value over time) was observed. The dynamics of changes for BMI, Cm, PA, and Z_200/5_ are presented in Figure 2 (A to D respectively). Detailed data regarding changes in BMI and selected BIA parameters in the subsequent months of observation are presented in Table 3.

### 3.2. The Correlation Between Selected Anthropometrical Parameters, BIA, and BMI in Measurement I

BMI was significantly positively correlated with all assessed anthropometric parameters. In turn, the Cm correlated positively with arm circumference (rho = 0.458, *p* = 0.0014), waist circumference (rho = 0.296, *p* = 0.0457), and WHR (rho = 0.348, *p* = 0.0279). A similar positive correlation was observed between the PA and biceps muscle skinfold (rho = 0.341, *p* = 0.0204), arm circumference (rho = 0.420, *p* = 0.0037), and WHR (rho = 0.366, *p* = 0.0221). In contrast, the Z200/5 parameter negatively correlated with biceps muscle skinfold (rho = −0.349, *p* = 0.0173), arm circumference (rho = −0.439, *p* = 0.0022), and WHR (rho = −0.375, *p* = 0.0170). Detailed data regarding the correlation between selected anthropometric parameters and BMI, Cm, PA 50 kHz, and Z_200/5_ values in the first measurement are presented in Table 4.

### 3.3. The Correlation between BMI and Selected BIA Parameters in the Subsequent Months of Observation

**Figure 3:**
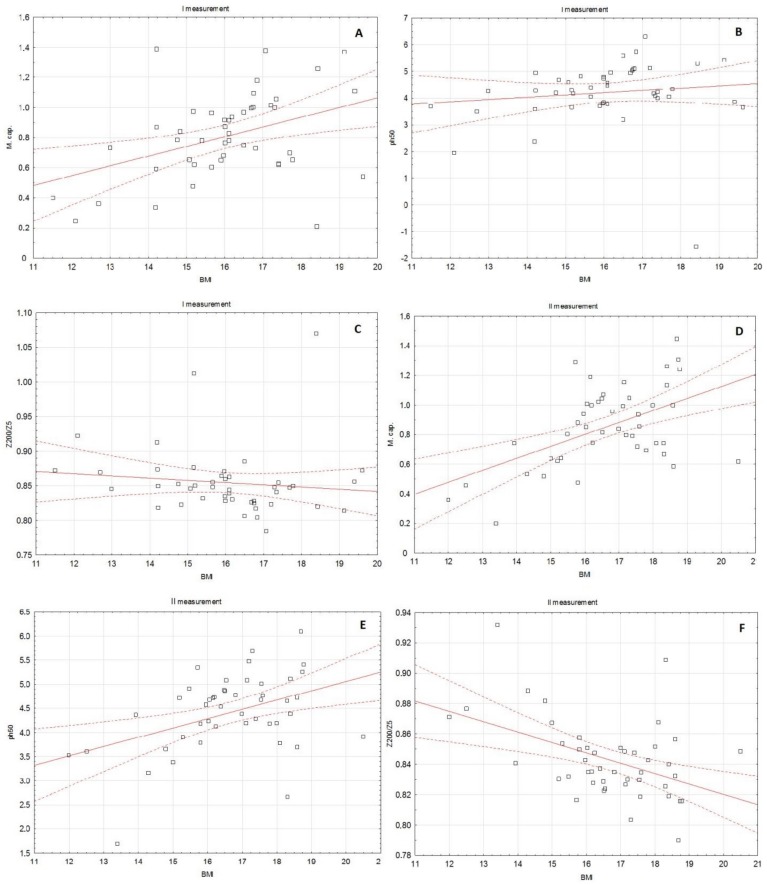
The correlation between BMI and the selected BIA parameters in the subsequent months of observation: BMI and membrane capacitance in I (**A**) and II (**D**) measurement, BMI and phase angle 50 kHz in I (**B**) and II measurement (**E**), BMI and Z_200/5_ in I (**C**) and II (**F**) measurement.

**Figure 4:**
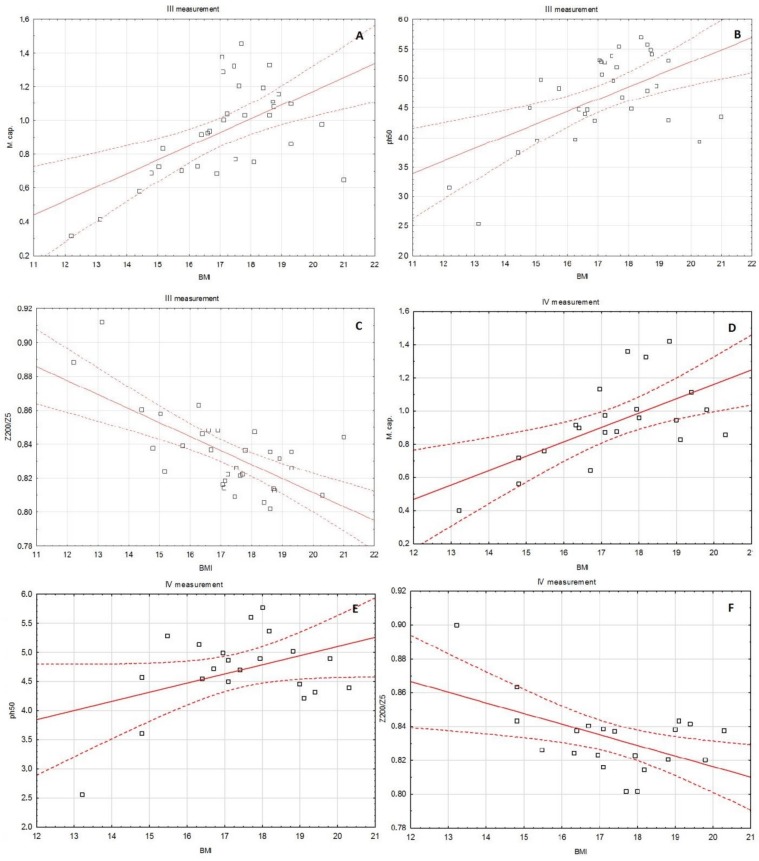
The correlation between BMI and the selected BIA parameters in the subsequent months of observation: BMI and membrane capacitance in III (**A**) and IV (**D**) measurement, BMI and phase angle 50 kHz in III (**B**) and IV measurement (**E**), BMI and Z_200/5_ in III (**C**) and IV (**F**) measurement.

In the first measurement, BMI significantly positively correlated with Cm (rho = 0.368, *p* = 0.00119) and PA (rho = 0.288, *p* = 0.0447). In contrast, the Z200/5 parameter negatively correlated with BMI (rho = −0.299, *p* = 0.0438). Similarly, in the second measurement, BMI significantly positively correlated with Cm (rho = 0.425, *p* = 0.0032) and PA (rho = 0.369, *p* = 0.0017), and negatively with Z_200/5_ (rho = −0.414, *p* = 0.0042). Also, in the third measurement, BMI significantly positively correlated with Cm (rho = 0.509, *p* = 0.0029) and PA (rho = 0.420, *p* = 0.0167), and negatively with Z_200/5_ (rho = −0.575, *p* = 0.0006). On the other hand, in the fourth measurement, BMI correlated only with Cm (positively, rho = 0.541, *p* = 0.0114). Detailed data about the correlation between BMI and selected BIA parameters in subsequent months of observation are presented in Table 5, Figure 3A–F, Figure 4A–F.

### 3.4. The Comparison of the Selected BIA Parameters Depending on the State of Malnourishment

The comparison of the selected BIA parameters (Cm, PA, and Z_200/5_) according to the state of starvation (according to DSM severe anorexia criteria and the extended BMI classification, BMI < 16.0 kg/m^2^) in the subsequent months of observation revealed that in all cases (Cm, PA, and Z_200/5_, except PA evaluated in the fourth measurement), their values were significantly different. In the case of Cm and PA, they were always lower in this state of malnourishment. On the other hand, Z_200/5_ was always higher in this group of patients. Detailed data from the comparison of selected BIA parameters dependent on the state of starvation are presented in Table 6.

### 3.5. The Usefulness of the Selected BIA Parameters in the Detection of the State of Starvation

**Figure 5:**
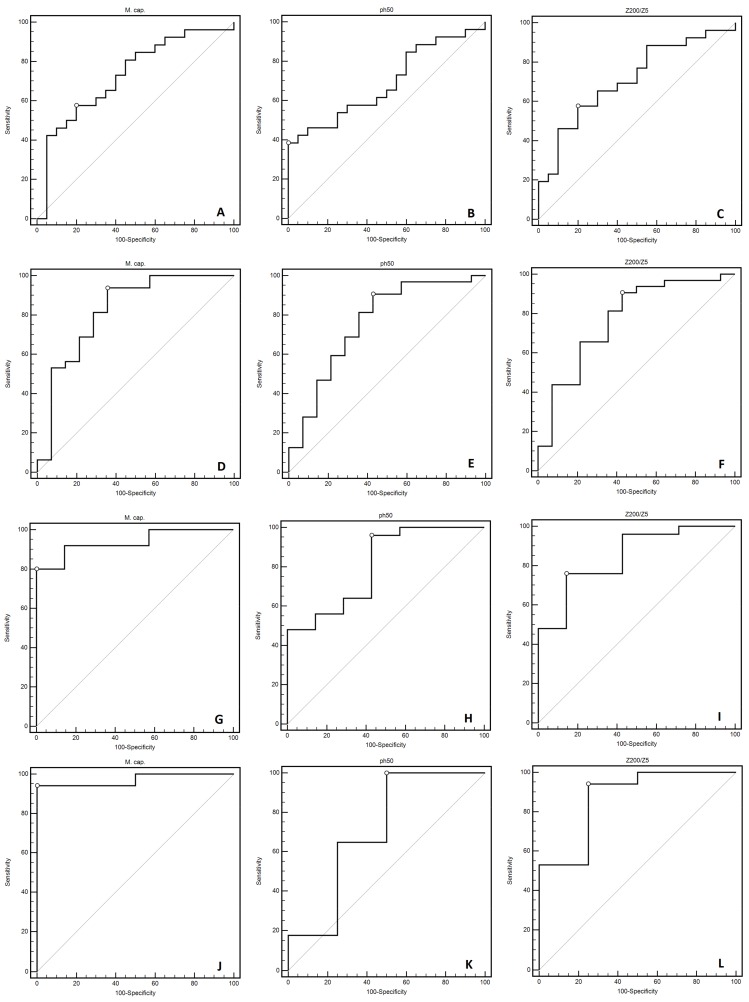
The ROC curve analysis used for the evaluation of the usefulness of the selected BIA parameters (membrane capacitance, phase angle 50 kHz, and Z_200/5_) in the detection of the state of starvation (BMI < 16 kg/m^2^) in the subsequent months of observation (measurements I (**A–C**), II (**D–F**), III (**G–I**), IV (**J–L**)).

We also evaluated the usefulness of the selected BIA parameters in the detection of the state of starvation (BMI < 16.0 kg/m^2^). ROC curve analysis revealed that all studied BIA parameters were characterized by statistically significant sensitivity and specificity in the detection of the state of starvation (the only exception was the PA in the fourth measurement) (Figure 5A–L). The ROC curve analysis results are presented in Table 7.

## 4. Discussion

Undernutrition present in AN is the reason for serious complications in the course of this disease, including death. Indeed, the main reported causes of deaths in anorexia nervosa are complications related to malnutrition, mainly heart failure, electrolyte disturbances, and suicide [29]. According to the ICD-10 and DSM 5, BMI is still the only recommended measurable parameter used for the physical assessment of AN patients [1,2]. However, it is important to identify potentially more accurate markers of malnourishment at the moment of AN diagnosis and then during the convalescence process.

### 4.1. Selected Anthropometrical Parameters, BIA, and BMI in Measurement I

In the first measurement, which was taken just after diagnosis, BMI was significantly positively correlated with all other assessed anthropometric parameters. This suggests that all anthropometric parameters deteriorate as they represent the external body condition. On the other hand, BIA is a method that deals with cells’ properties and does not assess only the external parts of the body. We found that BIA correlated significantly with only a few anthropometrical measurements. Cm, as the marker of cell membranes’ integrity, correlated positively with arm circumference, waist circumference, and WHR. A positive correlation was also discovered between PA and biceps muscle skinfold, arm circumference, and WHR. PA is a recognized nutritional status marker [7,8,9,10,11,12,13,14,15,16,17,18,19,20,21,22,23,24,25,26]. The Z200/5 parameter negatively correlated with biceps muscle skinfold, arm circumference, and WHR. Z_200/5_ is a parameter that negatively correlates with body condition [30]. It is worth noticing that all the BIA parameters used were correlated with WHR and arm circumference. As electrical parameters reflect the internal condition of the body, it has been suggested in other papers that they are more accurate for AN patients’ assessment than BMI [19,20,21,22,23,24,25,26]. On the other hand, it could also be interpreted that arm circumference and WHR are more reliable than other anthropometrical parameters. Fortunately, their measurements require only proper technique and body tape measure which is not expensive equipment for a clinical application. These changes could be also caused by nonsymmetrical abnormalities present in malnutrition. As shown in the study using segmental BIA during a therapy, the convalescence of patients does not take place in a symmetrical way for the whole body, but differs in different parts of the body [20]. Analogically, these changes could also be present at the moment of diagnosis.

### 4.2. The Changes of BMI and Selected BIA Parameters in the Subsequent Months of Observation

The changes of selected parameters in time were another aspect of this study. Significant changes were observed between the first, third, and fourth measurements in the case of BMI. The same tendency was noted in the case of selected BIA parameters (Cm, PA, and Z_200/5_). All of them were typical for convalescence as BMI, Cm, and PA values increased with time, and Z_200/5_ decreased. Although the changes of the parameters differed between follow-up stages, they were statistically significant. It could possibly be caused by individual response to the treatment by human subjects. A low PA value is indicative of diminished cellular integrity. Analogically, a higher PA value suggests larger quantities of intact cell membranes and thriving health. The role of PA in AN patients’ treatment assessment was reported in other works [21,22,23]. It was proven that it is a better discriminator between AN and constitutionally lean subjects than BMI and other anthropometrical measurements [24]. Changes of PA and reactance were also investigated in terms of metabolic changes in the course of AN patients’ convalescence. Both these parameters correlated with AN patients’ basal metabolic rate [25]. Furthermore, even though other studies have confirmed gradual changes of BMI and PA during AN patients’ treatment, it has been proven that in the case of recent convalescents, PA still differs from the population standards [23]. Over a long-term observation, PA can completely normalize, while BMI can still be outstanding compared to healthy subjects [21]. This study includes only four measurements and, correspondingly to the mentioned papers, shows PA and BMI increase over time [21,23]. Longer observation of enrolled subjects is suggested.

As mentioned above, at the beginning, BMI significantly positively correlated with Cm and PA, and negatively with Z_200/5_. The same correlation was observed in the second and third measurements. It is important that in the fourth measurement BMI correlated only with Cm. This change of the previous tendency can be explained when we realize that BMI provides information only about the total body mass and does not give proper information about, for example, fluid balance. Gaining weight could be the result of both fluid accumulation and soft tissue reconstruction. It was proven in the literature that the initial increase in the weight of the treated patients is due to the increase in extracellular water volume (after 3 weeks). During the continuation of treatment, an increase in intracellular water volume (after 3 months) was observed [22]. PA is a recognized nutritional marker that provides information about cell condition. Both BMI and PA increased in the last measurement, however they were not correlated at that time. Probably, the continuous BMI increase without PA correlation could be caused by stronger participation of fluid accumulation instead of soft tissue restitution at this level. However, it could be verified only with the application of bioelectrical impedance vector analysis.

### 4.3. Usefulness of Selected BIA Parameters in the Detection of the State of Starvation

All the applied BIA parameters turned out to be useful in the detection of malnutrition level. The ROC curve analysis demonstrated significant sensitivity and specificity of Cm, PA, and Z_200/5_ in the detection of the state of starvation. As mentioned above, the only exception was the PA in the fourth measurement, which was explained above as a possible result of fluid accumulation. BIA parameters turned out to be valuable not only in general condition assessment and monitoring of patients, but they can be also considered in terms of the malnutrition level. So far, there has only been one study that compared PA values depending on BMI value in AN patients [20]. It has confirmed that lower PA values were observed in patients with a BMI < 16.0 kg/m^2^. Furthermore, apart from this study, there are no other papers considering PA, Cm, or Z_200/5_ together or individually in the terms of severe AN. It is the first study that has taken all these BIA parameters into consideration and has proved that all of them are useful in the detection of the state of starvation.

### 4.4. Limitations of the Study

The study could possibly be extended with longer observation of the subjects until full recovery. Furthermore, even though it is the first study comparing all the mentioned anthropometrical and BIA parameters in AN patients, it could be extended to include more parameters in the future, for example biochemical parameters and psychiatric assessment results. As the purpose of the study was to investigate parameters during AN treatment, the healthy control group was not included. However, this is the subject of the other study.

## 5. Conclusions

This study shows a correlation between anthropometrical (circumferences of arm, calf, thigh, hips, waist, WHR, and skinfold test over biceps and triceps muscle, under the scapula, over the hip, 2 cm from the umbilicus) and BIA parameters (PA 50 kHz, Cm, and Z_200/5_). It is the first study that includes all these anthropometrical and BIA parameters. While all anthropometrical measurements were correlated with BMI, only WHR and arm circumference correlated with all BIA parameters. Afterwards, the follow-up performed in 6 week intervals revealed significant changes in BMI and BIA parameters during the recovery period (between first and third/fourth measurements, *p* < 005). They were all correlated until the fourth measurement, when BMI correlated only with Cm (*p* = 0.0114). It could be possibly caused by fluid accumulation; however, it could be verified only with bioelectrical impedance analysis vector application. In the mentioned literature it has been proven that BIA parameters are more adequate for nutritional status and treatment assessment in AN. The last part of the study has revealed that all the considered BIA parameters are useful in the detection of the state of starvation (*p* < 0.01).

## Figures and Tables

**Table 1:** Baseline characteristic of the study group.

Variable	*n* (%) or, Me ± SD (Range)
Sex–Female	46 (100%)
Age (years)	16 ± 4.99 (11–25)
Height (cm)	165.59 ± 6.00 (151–177)
Weight (kg)	44.02 ± 5.96 (34.20–57.30)
Fold biceps muscle (mm)	4.00 ± 2.56 (1.00–11.00)
Fold triceps muscle (mm)	7.50 ± 3.67 (1.00–18.00)
Fold under the scapula (mm)	6.00 ± 2.63 (1.00–14.00)
Fold over the hip (mm)	7.00 ± 3.57 (1.00–15.00)
Fold 2 cm from umbilicus (mm)	8.50 ± 4.91 (2.00–22.00)
Arm circumference (cm)	20.00 ± 2.27 (14.00–24.00)
Thigh circumference (cm)	42.00 ± 4.46 (30.00–52.00)
Calf circumference (cm)	30.00 ± 2.67 (24.50–38.50)
Waist circumference (cm)	60.00 ± 4.77 (50.00–73.00)
Hip circumference (cm)	83.00 ± 4.63 (72.00–93.00)
FM (kg)	8.70 ± 4.15 (3.34–25.46)
FM (%)	19.11 ± 7.63 (9.16–20.12)
FFM (kg)	35.47 ± 4.75 (21.34–46.76)
FFM (%)	80.89 ± 7.63 (45.60–90.84)
ICF (L)	14.12 ± 2.25 (6.25–18.65)
ICF (%)	54.77 ± 3.19 (40.04–59.72)
ECF (L)	11.74 ± 1.45 (9.16–15.58)
ECF (%)	45.23 ± 3.19 (40.28–59.96)
TBW (L)	25.97 ± 3.48 (15.62–34.23)
TBW (%)	59.21 ± 5.58 (33.38–66.49)
WHR ^1^	0.73 ± 0.03 (0.66–0.82)
BMI ^2^	16.10 ± 1.76 (11.50–19.61)
Cm ^3^	0.78 ± 0.28 (0.21–1.39)
PA (50 kHz) ^4^	4.27 ± 1.18 (1.56–4.20)
Z_200/5_ (200/5 kHz) ^5^	0.85 ± 0.05 (0.78–1.07)

^1^ WHR; waist-to-hip ratio, ^2^ BMI; body mass index, ^3^ Cm; membrane capacitance, ^4^ PA (50 kHz); phase angle measured at 50 kHz frequency, ^5^ Z200/5; Z200/5 parameter.

**Table 2:** The comparison of selected anthropometrical and BIA parameters according to the age of patients.

Variable	<18 Years Old, Me	>18 Years Old, Me	*p*
Height (cm)	164.50	169.00	0.0710
Weight (kg)	43.90	44.25	0.9448
Fold biceps muscle (mm)	4.00	3.50	0.5224
Fold triceps muscle (mm)	8.50	5.00	0.0225
Fold under the scapula (mm)	6.00	6.00	0.8430
Fold over the hip (mm)	7.00	5.50	0.2617
Fold 2 cm from umbilicus (mm)	9.50	7.00	0.1843
Arm circumference (cm)	20.25	19.00	0.1323
Thigh circumference (cm)	42.50	40.25	0.1138
Calf circumference (cm)	30.00	30.25	0.6595
Waist circumference (cm)	59.75	60.75	0.9448
Hip circumference (cm)	83.00	83.00	0.7730
FM (kg)	9.10	7.50	0.2216
FM (%)	19.99	17.32	0.2488
FFM (kg)	35.46	35.47	0.8536
FFM (%)	80.01	82.68	0.2488
ICF (L)	14.35	13.85	0.8717
ICF (%)	55.04	53.64	0.1462
ECF (L)	11.33	11.95	0.3102
ECF (%)	44.96	46.36	0.1462
TBW (L)	25.96	25.97	0.8536
TBW (%)	58.57	60.52	0.2488
WHR ^1^	0.73	0.73	0.8450
BMI ^2^	16.10	15.78	0.2088
Cm ^3^	0.89	0.65	0.0198
PA (50 kHz) ^4^	4.29	4.08	0.1736
Z_200/5_ (200/5 kHz) ^5^	0.84	0.85	0.1527

^1^ WHR; waist-to-hip ratio,^2^ BMI; body mass index, ^3^ Cm; membrane capacitance, ^4^ PA (50 kHz); phase angle measured at 50 kHz frequency, ^5^ Z200/5; Z200/5 parameter.

**Table 3:** Changes in BMI and selected BIA parameters in the subsequent months of observation.

Variable	Measurement				Significant Difference	*p*
	I	II	III	IV		
Weight (kg)	45.50	46.90	48.55	48.50	1 vs 3, 1 vs 4	0.0266
BMI ^1^	15.950	16.045	17.415	17.670	1 vs 3, 1 vs 4	0.0366
Cm ^2^	0.715	0.716	0.965	0.907	1 vs 3, 1 vs 4	0.0109
PA (50 kHz) ^3^	4.297	4.297	4.733	4.630	1 vs 3, 1 vs 4	0.0075
Z_200/5_^4^	0.847	0.842	0.836	0.837	1 vs 3, 1 vs 4	0.0297

^1^ BMI; body mass index, ^2^ Cm; membrane capacitance, ^3^ PA (50 kHz); phase angle measured at 50 kHz frequency, ^4^ Z200/5; Z200/5 parameter.

**Table 4:** Correlation between the selected anthropometrical, BMI, and BIA parameter values in measurement I.

Variable	Weight		BMI ^1^		Cm ^2^		PA 50 kHz ^3^		Z_200/5_^4^	
	Rho	*p*	Rho	*p*	Rho	*p*	Rho	*p*	Rho	*p*
Skinfold biceps muscle (mm)	0.610	<0.0001	0.608	<0.0001	0.260	0.0809	0.341	0.0204	−0.349	0.0173
Skinfold triceps muscle (mm)	0.613	<0.0001	0.647	<0.0001	0.175	0.2457	0.250	0.0940	−0.253	0.0894
Skinfold under the scapula (mm)	0.668	<0.0001	0.748	<0.0001	0.205	0.1709	0.156	0.3003	−0.157	0.2974
Skinfold over the hip (mm)	0.643	<0.0001	0.732	<0.0001	0.259	0.0824	0.202	0.1788	−0.224	0.1349
Skinfold 2 cm from umbilicus (mm)	0.511	0.0003	0.615	<0.0001	0.208	0.1664	0.202	0.1779	−0.226	0.1301
Arm circumference (cm)	0.692	<0.0001	0.845	<0.0001	0.458	0.0014	0.420	0.0037	−0.439	0.0022
Thigh circumference (cm)	0.872	<0.0001	0.842	<0.0001	0.274	0.0653	0.242	0.1051	−0.265	0.0754
Calf circumference (cm)	0.869	<0.0001	0.758	<0.0001	0.141	0.3496	0.098	0.5175	−0.146	0.3327
Waist circumference (cm)	0.786	<0.0001	0.748	<0.0001	0.296	0.0457	0.243	0.1044	−0.162	0.2806
Hip circumference (cm)	0.865	<0.0001	0.772	<0.0001	0.112	0.4569	0.045	0.7682	−0.003	0.9857
WHR ^5^	0.429	0.0057	0.430	0.0056	0.348	0.0279	0.361	0.0221	−0.375	0.0170

^1^ BMI; body mass index, ^2^ Cm; membrane capacitance, ^3^ PA 50 kHz; phase angle measured at 50 kHz frequency, ^4^ Z200/5; Z200/5 parameter, ^5^ WHR; waist-to-hip ratio.

**Table 5:** The correlation between BMI and BIA parameter values in the subsequent months of observation.

Measurement	Variable	Cm ^1^		PA 50 kHz ^2^		Z_200/5_^3^	
		Rho	*p*	Rho	*p*	Rho	*p*
I	BMI	0.368	0.0119	0.288	0.0447	−0.299	0.0438
II	BMI	0.425	0.0032	0.369	0.0117	−0.414	0.0042
III	BMI	0.509	0.0029	0.420	0.0167	−0.575	0.0006
IV	BMI	0.541	0.0114	0.053	0.8186	−0.337	0.1349
I	weight	0.175	0.2450	0.112	0.4577	−0.115	0.4470
II	weight	0.251	0.0922	0.148	0.3266	−0.208	0.1658
III	weight	0.303	0.0919	0.320	0.0741	−0.339	0.0573
IV	weight	0.227	0.3355	−0.048	0.8403	−0.171	0.4697

^1^ Cm; membrane capacitance, ^2^ PA 50 kHz; phase angle measured at 50 kHz frequency, ^3^ Z200/5; Z200/5 parameter.

**Table 6:** The comparison of the selected BIA parameters according to the state of starvation (BMI<16) in the subsequent months of observation.

Variable	BMI I Measurement, *Me*		*p*	BMI II Measurement, *Me*		*P*	BMI III Measurement, *Me*		*p*	BMI IV Measurement, *Me*		*p*
	<16	≥16		<16	≥16		<16	≥16		<16	≥16	
Cm ^1^	0.67	0.92	0.0084	0.63	0.97	0.0006	0.69	1.03	0.0005	0.64	0.96	0.0042
PA 50 kHz ^2^	4.17	4.52	0.0299	3.84	4.69	0.0056	3.94	4.95	0.0147	4.09	4.87	0.2099
Z_200/5_^3^	0.85	0.84	0.0148	0.85	0.83	0.0039	0.86	0.83	0.0058	0.85	0.82	0.0252

^1^ Cm; membrane capacitance, ^2^ PA 50 kHz; phase angle measured at 50 kHz frequency, ^3^ Z_200/5_; Z_200/5_ parameter.

**Table 7:** The ROC curve analysis of the usefulness of the selected BIA parameters in the detection of the state of starvation (BMI < 16 kg/m^2^) in the subsequent months of observation.

Variable	I Measurement	II Measurement	III Measurement	IV Measurement
**Cm ^1^**				
cut-off	>0.87	>0.64	>0.83	>0.76
Sensitivity	57.69	93.75	80	94.12
Specificity	80	64.29	100	100
AUC (95%CI)	0.73 (0.58–0.85)	0.82 (0.68–0.92)	0.94 (0.79–0.99)	0.97 (0.79–1.00)
*p*	0.0029	<0.0001	<0.0001	<0.0001
**PA 50 kHz ^2^**				
cut-off	>4.93	>3.90	>3.94	>3.61
Sensitivity	38.46	90.62	96.00	100
Specificity	100	57.14	47.14	50
AUC (95%CI)	0.69 (0.53–0.82)	0.76 (0.61–0.87)	0.81 (0.63–0.98)	0.71 (0.47–0.88)
*p*	0.0164	0.0023	0.0018	0.3033
**Z_200/5_^3^**				
cut-off	≤0.84	≤0.85	≤0.84	≤0.84
Sensitivity	57.69	90.62	76.00	94.12
Specificity	80	57.14	85.71	75.00
AUC (95%CI)	0.71 (0.56–0.83)	0.77 (0.62–0.88)	0.85 (0.67–0.95)	0.87 (0.65–0.97)
*p*	0.0062	0.0009	<0.0001	0.0021

^1^ Cm; membrane capacitance, ^2^ PA 50 kHz; phase angle measured at 50 kHz frequency, ^3^ Z200/5; Z200/5 parameter.
